# Interleukin-1 beta and neurotrophin-3 synergistically promote neurite growth *in vitro*

**DOI:** 10.1186/1742-2094-8-183

**Published:** 2011-12-26

**Authors:** Francesco Boato, Daniel Hechler, Karen Rosenberger, Doreen Lüdecke, Eva M Peters, Robert Nitsch, Sven Hendrix

**Affiliations:** 1 Dept. of Functional Morphology & BIOMED Institute, Hasselt University, Belgium; 2 Current address: Institut de la Vision, Université Pierre et Marie Curie, Paris, France; 3 Institute of Cell Biology and Neurobiology, Center for Anatomy, Charité - Universitätsmedizin Berlin, Germany; 4 Psychoneuroimmunology, University-Medicine Charité, Charité Center 12 for Internal Medicine and Dermatology, D-10117 Berlin, Germany; 5 Department of Psychosomatic Medicine, Justus-Liebig-University, Gießen, Germany; 6 Institute for Microscopic Anatomy and Neurobiology, University Medicine Mainz, Johannes Gutenberg University Mainz, Germany

**Keywords:** interleukin 1 beta, IL-1β, neurotrophin 3, NT-3, NGF, spinal cord, brain slices, neurite growth, axon outgrowth, neuroplasticity

## Abstract

Pro-inflammatory cytokines such as interleukin-1 beta (IL-1β) are considered to exert detrimental effects during brain trauma and in neurodegenerative disorders. Consistently, it has been demonstrated that IL-1β suppresses neurotrophin-mediated neuronal cell survival rendering neurons vulnerable to degeneration. Since neurotrophins are also well known to strongly influence axonal plasticity, we investigated here whether IL-1β has a similar negative impact on neurite growth. We analyzed neurite density and length of organotypic brain and spinal cord slice cultures under the influence of the neurotrophins NGF, BDNF, NT-3 and NT-4. In brain slices, only NT-3 significantly promoted neurite density and length. Surprisingly, a similar increase of neurite growth was induced by IL-1β. Additionally, both factors increased the number of brain slices displaying maximal neurite growth. Furthermore, the co-administration of IL-1β and NT-3 significantly increased the number of brain slices displaying maximal neurite growth compared to single treatments. These data indicate that these two factors synergistically stimulate two distinct aspects of neurite outgrowth, namely neurite density and neurite length from acute organotypic brain slices.

## Introduction

Interleukin-1 beta (IL-1β) is a member of the IL-1 family of cytokines which have potent pro-inflammatory properties. It is produced in the periphery mainly by monocytes and is a strong activator of the host immune response to both injury and infection [1,2]. In the central nervous system (CNS) IL-1β is primarily produced by microglia and invading monocytes/macrophages, but other types of resident cells of the nervous system, including neurons and astrocytes, are also capable of its production [3]. It is generally believed that inflammatory processes stimulated by pro-inflammatory cytokines and particularly by IL-1β, are rather detrimental and can aggravate the primary damage caused by infection of the CNS. This has been suggested by various *in vivo *studies, in line with its enhanced expression in the brain after damage or in neurodegenerative diseases, including Alzheimer's disease (AD). Consistently, IL-1 deficient mice display reduced neuronal loss and infarct volumes after ischemic brain damage [4] and direct application of the recombinant cytokine results in an enhanced infarct volume [5]. In traumatic brain injury, antibodies against IL-1β reduce the loss of hippocampal neurons [6]. Consistently, in a mouse model of AD, an inhibitor of pro-inflammatory cytokine production suppressed neuroinflammation leading to a restoration of hippocampal synaptic dysfunction markers [7]. In AD it has also been demonstrated that members of the IL-1 family are associated with an increased risk of contracting the disease [8].

The findings in various *in vitro *models suggest a rather elaborated mechanism. In culture, IL-1β demonstrated neurotoxic effects towards hippocampal neurons exposed to high concentrations (500 ng/ml) combined with long-term exposure (three days). However, no effect was observed in lower concentrations following short-term exposure (one day) [9]. In other *in vitro *models, IL-1β has even been seen to display beneficial effects towards neuronal survival in the CNS [10,11]. This has also been observed in axonal growth in the peripheral nervous system both *in vivo *following sciatic nerve injury [12,13] and *in vitro *in adult dorsal root ganglion (DRG) collagen gel explant cultures [14], but not in dissociated single DRG neuron cultures [15].

Previously, it has been demonstrated that IL-1β impairs neurotrophin-induced neuronal cell survival [16,17]. It has long been hypothesized that cytokine effects on neurite growth may be mediated at least in part by modulating neurotrophin signalling accordingly [18]. In addition to their positive effect on cell death, the neurotrophins Nerve Growth Factor (NGF), Brain-derived Neurotrophic Factor (BDNF), Neurotrophin-3 (NT-3) and NT-4 have also a well documented impact on axon plasticity and regeneration [19,20]. This is crucial in the context of CNS insult to provide re-innervation and thus consecutive functional recovery. Based on these observations we investigated whether IL-1β is also a modulator of neurotrophin-induced neurite outgrowth in the CNS *in vitro*, using organotypic brain and spinal cord slice cultures. The present study shows that surprisingly, IL-1β did not abrogate NT-3-induced neurite outgrowth but conversely showed a significant synergistic effect. These data indicate that IL-1β differentially regulates the effect of NT-3 on neuronal survival and neurite extension.

## Materials and methods

### Animals and factors

C57BL/6 wildtype mice and IL-1β-deficient mice [21] were housed in a conventional animal facility (Center for Anatomy, Charité-Universitätsmedizin, Berlin, Germany). All experiments were performed in accordance with German guidelines on the use of laboratory animals. Recombinant neurotrophins NGF, BDNF, NT-3 and NT-4 were used in a concentration of 500 ng/mL (all Tebu-Bio, Offenbach, Germany). Recombinant IL-1β (Tebu-Bio, Offenbach, Germany) was used in concentrations of 5, 50 and 500 ng/mL.

### Acute organotypic brain slice culture

The entorhinal slice cultures were prepared from mouse brains at postnatal day 2 as previously described [22-25]. In brief, after decapitation, the entorhinal cortex was dissected in ice-cold preparation medium, containing MEM with L-Glutamine (2 mM) and Trisbase (8 mM). Transverse slices 350 μm thick were cut using a tissue chopper (Bachhofer, Reutlingen, Germany). Collagen was prepared as previously described [26]. Each entorhinal slice was embedded in a drop of collagen matrix on glass slides. The recombinant factors (neurotrophins and IL-1β) were mixed into the sterile cultivation medium containing MEM, 25% HBSS, 25% heat-inactivated normal horse serum, 4 mM L-glutamine, 4 μg/ml insulin (all from Gibco, Karlsruhe, Germany), 2.4 mg/ml glucose (Braun, Melsungen, Germany), 0.1 mg/ml streptomycin, 100 U/ml penicillin, and 800 μg/ml vitamin C (all Sigma-Aldrich, Taufkirchen, Germany). The collagen co-cultures were incubated at 37°C in a humidified atmosphere with 5% CO_2_. After 48 h *in vitro*, the collagen slices were analyzed microscopically (Olympus IX70, Hamburg, Germany).

Neurotrophin concentrations were chosen after extensive pilot experiments based on studies by the Kapfhammer group on age-dependent regeneration of entorhinal fibers in mouse slice cultures [19], which showed that substantially higher concentrations are needed for brain slices compared to primary cell cultures.

### Measurement of axonal density and length of organotypic brain slice cultures

To evaluate the axon outgrowth from entorhinal cortex explants, we improved a pragmatic, reliable and reproducible method, with which the axonal density and length was evaluated after two days in culture [23,27]. Two independent blinded investigators evaluated neurite density on a scale from 0 (no axons) to 3 (multiple axons), at a total magnification of 200, using a 20× Olympus LCPLANFL objective (Olympus IX70, Hamburg, Germany). Axonal length was quantified at a total magnification of 100, using a 10× Olympus LCPLANFL objective and a widefield eyepiece with a grid of 100 × 100 μm (Olympus WH 10X2-H, Hamburg, Germany) and by measuring the length of a minimum of 10 axons growing in the same direction and reaching the same length: grade 0 (0 - 200 μm), 1 (200 - 400 μm), 2 (400 - 800 μm) and 3 (> 800 μm). Slices with a score equal 3 in length or density, where considered as having "maximum growth" and were then used for further analysis. For combined "maximum density and length" analysis, only the slices which reached the maximum score in both parameters were selected. All experiments were repeated at least three times.

### Acute organotypic transverse spinal cord slice cultures

Transverse spinal cord cultures were prepared from mice at embryonic stage 13 (E13). After preparation out of the amniotic sac, embryos were decapitated and skin and organs were removed to isolate the spinal column, it was immediately transferred into ice cold HBSS medium. After dissection of the spinal cord, the remaining dorsal root ganglia (DRG) were removed and lumbar and cervical spinal sections dismissed. The thoracic segment was cut with a tissue chopper into 350 μm slices. These slices were divided along the sulcus medianus into two halves and each placed into a drop of collagen (as described above) with the cut surface of the sulcus medianus showing upwards. After polymerization of the collagen, 500 μl of medium with or without factors were added to the slices. The transverse spinal cord slices were incubated at 37°C in a humidified atmosphere with 5% CO_2_. After 48 h *in vitro*, the collagen slices were analyzed microscopically (Olympus IX70, Hamburg, Germany).

### Measurement of axonal outgrowth from transverse spinal cord slices

Axonal outgrowth of the transverse spinal cord slices was evaluated as described previously for organotypic dorsal root ganglia cultures [28]. Slices were photographed in PBS with two fixed exposure times to visualize the neurite area and the slice, respectively. The ratio between these two areas was calculated and matched between slices with or without factor. All experiments were repeated at least three times.

### Statistical analysis

The results are expressed as mean ± SEM. The values from the experimental cultures were compared to control cultures prepared in the same experiment (double treatment with NT-3 and IL-1β were additionally compared to single treatments). Subsequently, the data of each group were pooled for statistical analysis. After confirming that significant differences existed between the various groups by performing a Kruskal-Wallis Test, p-values were determined, using a Mann-Whitney-U test. A Chi^2^-test was used to test if the frequency of maximal neurite growth was significantly different between the groups.

## Results

**Figure 1:**
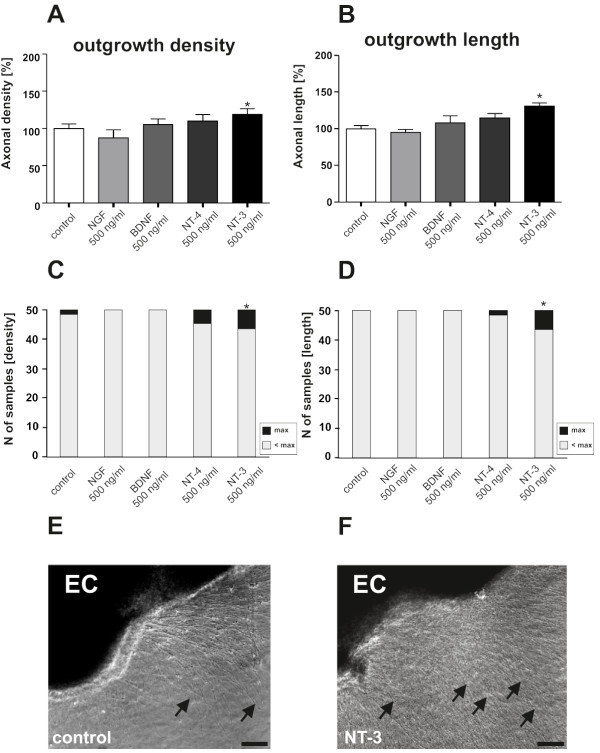
**Recombinant NT-3 stimulates neurite density and length of organotypic brain slices**. The neurotrophins NGF, BDNF, NT-3 and NT-4 (500 ng/ml) were added to the culture medium immediately after preparation of the organotypic brain slices. NT-3, but not the other neurotrophins significantly increases neurite density (A), neurite length (B), the amount of slices reaching the maximum outgrowth (C) and the amount of slices reaching the maximum length (D). E + F: representative photomicrograph showing the increase in outgrowth of NT-3 treated EC slices compared to control. n = 50 slices. A + B: *: Statistically significant difference to control; p < 0.05 (Mann Whitney U test). C + D: *: Statistically significant difference; p < 0.05 (Chi-square analysis). EC = enthorinal cortex. Arrows indicate outgrowing neuritis. Scale bar: 100 μm.

Previously, IL-1β has been described as a negative modulator of neurotrophin-induced neuronal survival [16,17]. Therefore, we investigated whether IL-1β has a similar negative impact on NT-3-induced neurite growth from organotypic brain slices and transverse spinal cord slices. As a first step we investigated the effects of different neurotrophins on neurite growth in a classical model of organotypic brain slice cultures. Organotypic brain slices were embedded in a three-dimensional collagen matrix in the presence of 500 ng/mL NGF, BDNF, NT-3 or NT-4 or solvent. These concentrations were chosen after extensive pilot studies based on the landmark studies by the Kapfhammer group on regeneration of entorhinal fibers in murine slice cultures [19]. Neurite density and length was microscopically analyzed (Figure 1). Compared to control brain slices, neurite density was significantly increased by about 20% after cultivating with NT-3. It is important to note that an increase of 20% is close to the maximum increase of axon outgrowth which can be induced in brain slices with our method of analysis.

Such an increase is not seen after administration of the other neurotrophins (Figure 1A).

Similarly, NT-3 also significantly increased the length of the cortical neurites when compared to untreated controls while the other neurotrophins had no effect on neurite length (Figure 1B). Thus, only recombinant NT-3 (but not NGF, BDNF or NT-4) is capable of stimulating neurite outgrowth as well as neurite length from entorhinal cortical neurons (Figure 1E, F). A Chi^2 ^test also revealed a significant increase in the number of slices reaching maximal neurite density and length in the presence of NT-3, compared to untreated controls (Figure 1C, D).

**Figure 2:**
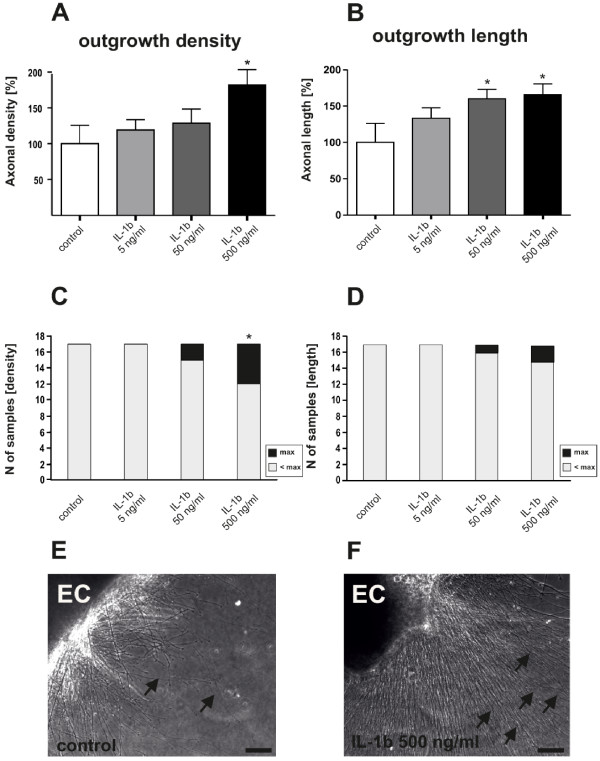
**IL-1β stimulates neurite density and length in organotypic brain slices**. A dose-response curve revealed that high doses of IL-1β (500 ng/ml) added to the culture medium, stimulate neurite density (A) of organotypic brain slices and the amount of slices reaching the maximum outgrowth (C). A lower dose (50 ng/ml) is still able to stimulate the average length of neuritis (B) but neither 500 ng/ml nor 50 ng/ml of IL-1β were able to significantly increase the amount of slices presenting maximum length (D). E + F: representative photomicrograph showing the increase in outgrowth of IL-1β treated EC compared to control. n = 17 slices. A + B: *: Statistically significant difference to control; p < 0.05 (Mann Whitney U test). C + D: *: Statistically significant difference; p < 0.05 (Chi-square analysis) EC = enthorinal cortex. Arrows indicate neuritis. Scale bar: 100 μm.

Since the effect of the inflammation-associated cytokine IL-1β on repair mechanisms in the CNS is controversial, we analyzed as a second step IL-1β effects on neurite growth from organotypic brain slices by adding it to the medium in three different concentrations (5, 50 and 500 ng/ml) (Figure 2). The highest concentration of IL-1β significantly stimulated and nearly doubled neurite density compared to control treated slices (Figure 2A, E, F). Neurite elongation was significantly increased by 50 and 500 ng/ml of IL-1β (Figure 2B). Moreover, the Chi^2 ^test showed a significant increase in the number of slices displaying maximal neurite density in the presence of 500 ng/ml IL-1β, compared to untreated controls (Figure 2C, D).

**Figure 3:**
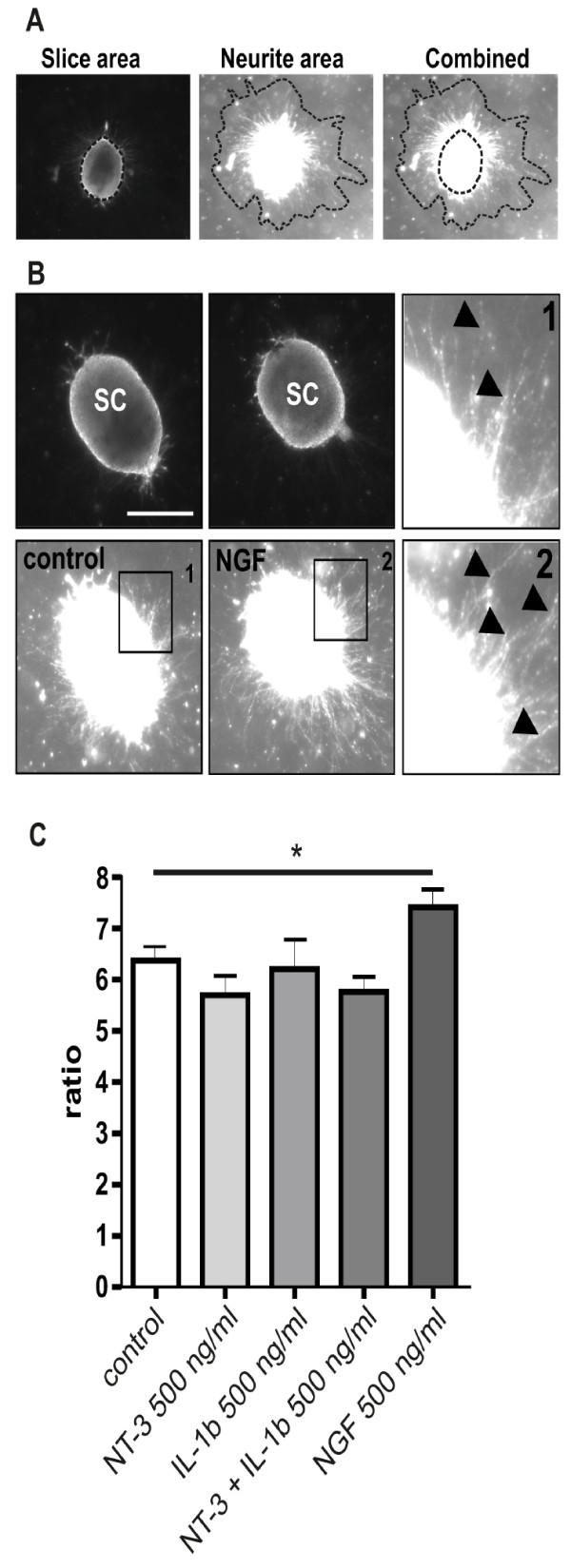
**NT-3 and IL-1beta do not increase neurite outgrowth of transverse spinal cord slices**. A: Transverse spinal cord slices were prepared from E13 spinal cords and the ratio between neurite area and slice area were compared. B: representative photomicrograph showing the increase in outgrowth of NGF treated EC compared to control. NGF (500 ng/ml) serves as positive control. C: NT-3 and IL-1β (500 ng/ml) were added to the culture medium of organotypic transverse spinal cord slices. Only NGF significantly increases neurite density, while NT-3, IL-1β or a combination of these factors does not influence neurite outgrowth. n = 9-11 slices. *: Statistically significant difference to control; p < 0.05 (Mann Whitney U test). Arrow heads indicate outgrowing neuritis. Scale bar: 50 μm.

In order to investigate potential differences between the effects of IL-1β and NT-3 on cerebral and spinal cord neurites, we further analyzed both factors in a model of organotypic transverse spinal cord slices (Figure 3). Spinal cord slices were embedded in a collagen matrix similar to the brain slice model and the ratio between outgrowth area and slice size was determined (Figure 3A, see materials & methods section for details). Surprisingly, the application of 500 ng/ml of NT-3 or IL-1β as well as the combined application of both factors at the same concentration, had no effect on the outgrowth ratio compared to control slices, suggesting a cortex-specific effect of both factors (Figure 3C). As a positive control for the model we used 500 ng/ml of NGF, which significantly stimulated the outgrowth ratio of transverse spinal cord slices compared to untreated controls (Figure 3B, C).

**Figure 4:**
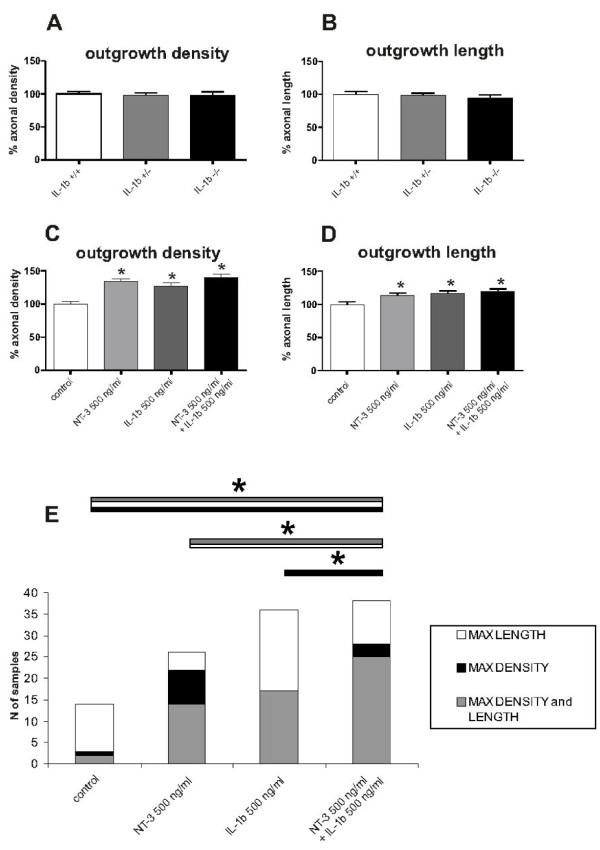
**Neurite outgrowth is independent on endogenous IL-1beta and is synergistically stimulated by combined application of NT-3 and IL-1beta**. A + B: Neurite outgrowth (neurite density A and neurite length B) was not influenced in the absence of endogenous IL-1β in IL-1β-deficient mice. Heterozygous IL-1β-deficient and wildtype mice served as controls. n: 50 slices. C + D: The combined administration of NT-3 and IL-1β shows only a slight increase in neurite density and length, if compared to single treatments. n = 84 slices. *: Statistically significant difference to control; p < 0.05 (Mann Whitney U test). Error bars represent SEM. E: Chi-square analysis reveals a significant difference in the frequency of brain slices with maximal outgrowth between single treatments with NT3 or IL-1β and the combined administration of both factors. Particularly double treatment presents a significant increase in the number of slices reaching maximum density if compared to control and single treatment with IL-1β, and in length if compared to single treatment with NT3. Additionally, combination of the two factors is also characterized by a significantly higher number of slices which hit the maximum in both parameters, if compared to control condition or single treatment with NT3 (to increase the readability of the graph only the significances relative to the double treatment have been included). *: Statistically significant difference; p < 0.05 (Chi-square).

The importance of endogenous IL-1β on spontaneous neurite growth from organotypic brain slices was then determined by cultivating slices from IL-1β knock out mice (Figure 4A and 4B). We compared the neurite density and neurite length from wildtype animals with heterozygous and homozygous IL-1β-deficient animals, all derived from the same litter and differentiated by PCR after evaluating the experiments. We found no significant difference between the groups; thus, neurite density as well as neurite length of organotypic brain slices is independent of *endogenous *IL-1β.

To elucidate whether IL-1β has a suppressive effect not only on neurotrophin-induced neuron survival, but also on neurite growth we co-administrated IL-1β and NT3 to acute brain slices (Figure 4C-E). As shown in Figure 1 and 2, both factors alone stimulated neurite density and extension from organotypic brain slices and the combined administration of IL-1β and NT-3 (both 500 ng/ml) could not further promote the mean neurite density and neurite length (Figure 4C, D). However, the Chi^2 ^test showed that the combination of both factors resulted in a significantly higher number of slices reaching maximal neurite density compared to controls and slices treated only with IL-1β. Additionally, the combination of both factors exerts a similar effect on maximal neurite length when compared to controls and slices treated only with NT-3. Finally, a significantly higher number of slices treated with both factors reached maximal levels of both parameters, i. e. combined maximal density and length, when compared to control and NT-3 treated slices (Figure 4E). Thus, the combined application of NT-3 and IL-1β allowed higher numbers of slices to reach maximum values of density and/or length which was not achieved by the application of the single factors.

In summary, IL-1β promotes increased neurite density and length from organotypic brain slices and does not inhibit NT-3-induced neurite growth, but conversely, it shows a synergistic effect in contrast to its suppressive effect on NT-3-induced neuronal survival [16,17].

## Discussion

Interleukin-1 beta (IL-1β) is a pluripotent cytokine and a main component of many inflammatory pathways. It is overexpressed after central nervous system (CNS) insult, primarily by microglia and macrophages, as part of the local tissue reaction [3,29,30]. Increased levels of the cytokine are documented both in chronic neurodegenerative disease and after acute mechanical injury. To examine its effect on neurodegeneration, studies have focused mainly over the last two decades, on Alzheimer's disease (AD) [31]. Elevated plasma levels of IL-1 had been reported in patients with AD (almost 40-fold higher than in the healthy brain)[32] and there is evidence of a correlation between IL-1β gene polymorphism and the risk of contracting the disease [33,34]. It is currently under investigation as a marker of ongoing brain neurodegeneration, even though levels are also elevated in the healthy aging brain [35]. In line with the documented negative effect on survival, it has been demonstrated that IL-1β impairs NT-3- and BDNF-mediated trophic support of cortical neurons by interfering with the Akt and MAPK/ERK intracellular pathway [16,17], therefore abrogating their neuroprotective properties.

However, there is increasing evidence that inflammation-associated cytokines can play a key role in stimulating neurite growth and regeneration [18,36]. As mentioned before, aside from neurodegenerative diseases, IL-1β levels are elevated after mechanical damage to the CNS. Notoriously after mechanical damage in the CNS, two major events occur that slow down or even inhibit regenerative processes. The first is the secondary damage of primarily unharmed neurons, with the second being the intrinsic inhibition of neurite plasticity and reestablishment of a proper neurite network [37-39]. Pro-inflammatory cytokines produced after mechanical damage to the CNS are considered as being negative for neuronal survival and regeneration [40]. However, the role of IL-1β is still controversial, with conflicting *in vivo *and *in vitro *data published in the literature [40]. To our knowledge - there is very little literature about the role of IL-1β in axon regeneration in the CNS. In contrast, there is extensive literature about the implication of the neurotrophins Nerve Growth Factor (NGF), Brain-derived Neurotrophic Factor (BDNF), Neurotrophin-3 (NT-3) and NT-4, in traumatic CNS lesions. These are well known for their neuroprotective effects as well as their ability to promote neurite growth via independent mechanisms [41-44]. The focus of the present study was then to outline whether IL-1β is also able to abrogate neutrophin-induced effects on CNS plasticity, as shown for neutrophin-dependent trophic support for neuronal cell survival.

We started our study by investigating the effect of neurotrophins in a well established model of outgrowth from organotypic brain slices. Surprisingly, only recombinant NT-3 (but not NGF, BDNF or NT-4) was able to stimulate neurite outgrowth as well as neurite length from organotypic brain slices, also increasing the number of slices displaying maximal outgrowth. This is in contrast to several single cell studies in which neurotrophins are highly efficient in promoting axonal growth [45-47]. However, brain slices should be considered as an organotypic model of brain trauma, and therefore appear to be closer to the *in vivo *situation than single cell cultures [48-50], since the organotypic environment of neurons is composed of astrocytes, microglia cells and other immune cells [25,51,52].

Interestingly, we also showed that administration of IL-1β at varying concentrations to the brain slices lead to a significant increase in density and neurite length, when compared to untreated control slices. Key effects of IL-1β in this context include the induction of IL-6, tumor necrosis factor (TNF)-α and nitric oxide [53] and increased proliferation of macrophages [54] and astrocytes [55-57]*in vitro *and *in vivo*. Both IL-6 and TNF-α are associated with stimulating properties of neurite growth. It was demonstrated that TNF-α can support glia-dependent neurite growth in organotypic mesencephalic brain slices [58] and is a key factor in the hypothermia induced neurite outgrowth, also as a recombinant factor [24]. The neuropoietic cytokine IL-6 is known to be a potent stimulating factor of neurite growth and regeneration in organotypic hippocampal slices [59] as well as in dorsal root ganglion cells [28]. Furthermore, IL-1β is capable of activating the production of growth factors in CNS-derived cells. It induces NGF [60-62], fibroblast growth factor (FGF)-2 and S100B production from astrocytes. FGF-2 can be a trophic factor for motor neurons or basal forebrain neurons [63,64] and IL-1β-induced S100B overexpression is likely to be responsible for the excessive growth of dystrophic neuritis in AD plaques [65]. It was also demonstrated that IL-1β can promote neurite outgrowth from DRGs and cerebellar granule neurons (CGNs) by deactivating the myelin-associated glycoprotein (MAG) RhoA pathway via p38 MAPK activation [12,13].

In the spinal cord, IL-1β has been implicated in extensive inflammation and progressive neurodegeneration after ischemic and traumatic injury [66,67]. That is supported by the finding that administration of an IL-1 receptor antagonist reduced both neuronal necrosis and apoptosis in a model of spinal cord ischemic-reperfusion injury in rabbits [68]. Since IL-1β had the capacity to stimulate neurite growth in brain slices, we tested if the same effect could be achieved in a *de novo *organotypic spinal cord slice model. Surprisingly neither the single administration of IL-1β or NT-3, nor the combined administration of both factors had an influence on the measured neurite growth from the spinal cord slices. These findings may suggest that potent NT-3 effects on neuronal regeneration in the injured spinal cord [69-71] are not the result of modulating segmental spinal cord neurons but rather direct or indirect effects on axons deriving from the motorcortex.

Another difference from the brain situation is that NGF had a stimulating effect on neurite outgrowth from the spinal cord slices which was not present in the entorhinal cortex. This might be due to the time and location dependent regulation of the Trk receptors, influencing the effectiveness of the neurotrophins [72,73].

As described above, in 2008 the Cotman group presented two publications demonstrating that IL-1β is a negative regulator of neuronal survival, due to its interference with the trophic signalling of NT-3 and BDNF. Previous work of our group indicated that neuronal survival and neurite growth can be two independent phenomena; e.g. while hypothermia has a negative effect on the neuronal survival [74], we demonstrated that in the same conditions neurite outgrowth is substantially increased and is dependent on tumor necrosis factor (TNF)-α [24]. To test the effect of IL-1β on NT-3-induced neurite growth, we applied both factors on enthorinal cortex slices. Interestingly, even without evident further stimulation in mean density and length compared to the single administration, a Chi square analysis revealed that the double administration leads to a significantly higher number of slices reaching the maximum level of outgrowth (density or length), when compared to the single treatments.

In conclusion, our results demonstrate that NT-3, but not the other neurotrophins, can stimulate neurite growth in organotypic brain slices. In contrast, neither NT-3 nor IL-1β are capable of enhancing neurite growth from spinal cord slices. Furthermore, we were able to demonstrate that the pro-inflammatory cytokine IL-1β has a positive effect on neurite growth from cortical slices and does not abolish the stimulating effect of NT-3, having instead a synergistic effect. As a result anti-inflammatory treatments for AD or mechanical brain damage may have a positive effect on neuronal cell death, with the risk of limiting neurite regrowth.

## Competing interests

The authors declare that they have no competing interests.

## Authors' contributions

FB participated in the analysis of the data, the preparation of the figures and wrote the manuscript, DH performed the brain slices experiments, analyzed the data and contributed in the drafting of the manuscript. KR performed the spinal cord slices experiments and analyzed the data. DL participated in performing the experiments and analyzing the data. EMP participated in the analysis of the data. RN contributed in conceiving the study and providing research support. SH conceived the study, participated in its design, provided research support and wrote the manuscript. All authors read and approved the final version of the manuscript.

## References

[B1] Peschke T Bender A Nain M Gemsa D Role of macrophage cytokines in influenza A virus infections Immunobiology 1993 189 3-4 340 355 10.1016/S0171-2985(11)80365-7 8125516

[B2] Hildebrand F Pape HC Krettek C The importance of cytokines in the posttraumatic inflammatory reaction Unfallchirurg 2005 108 10 793 794, 796-803 10.1007/s00113-005-1005-1 16175346

[B3] Bauer J Berkenbosch F Van Dam AM Dijkstra CD Demonstration of interleukin-1 beta in Lewis rat brain during experimental allergic encephalomyelitis by immunocytochemistry at the light and ultrastructural level Journal of neuroimmunology 1993 48 1 13 21 10.1016/0165-5728(93)90053-2 8227304

[B4] Boutin H LeFeuvre RA Horai R Asano M Iwakura Y Rothwell NJ Role of IL-1alpha and IL-1beta in ischemic brain damage J Neurosci 2001 21 15 5528 5534 11466424 10.1523/JNEUROSCI.21-15-05528.2001 PMC6762680

[B5] Loddick SA Rothwell NJ Neuroprotective effects of human recombinant interleukin-1 receptor antagonist in focal cerebral ischaemia in the rat J Cereb Blood Flow Metab 1996 16 5 932 940 8784237 10.1097/00004647-199609000-00017

[B6] Lu KT Wang YW Yang JT Yang YL Chen HI Effect of interleukin-1 on traumatic brain injury-induced damage to hippocampal neurons J Neurotrauma 2005 22 8 885 895 10.1089/neu.2005.22.885 16083355

[B7] Ralay Ranaivo H Craft JM Hu W Guo L Wing LK Van Eldik LJ Watterson DM Glia as a therapeutic target: selective suppression of human amyloid-beta-induced upregulation of brain proinflammatory cytokine production attenuates neurodegeneration J Neurosci 2006 26 2 662 670 10.1523/JNEUROSCI.4652-05.2006 16407564 PMC6674428

[B8] Grimaldi LM Casadei VM Ferri C Veglia F Licastro F Annoni G Biunno I De Bellis G Sorbi S Mariani C Association of early-onset Alzheimer's disease with an interleukin-1alpha gene polymorphism Ann Neurol 2000 47 3 361 365 10.1002/1531-8249(200003)47:3<361::AID-ANA12>3.0.CO;2-N 10716256

[B9] Araujo DM Cotman CW Differential effects of interleukin-1 beta and interleukin-2 on glia and hippocampal neurons in culture Int J Dev Neurosci 1995 13 3-4 201 212 10.1016/0736-5748(94)00072-B 7572276

[B10] Carlson NG Wieggel WA Chen J Bacchi A Rogers SW Gahring LC Inflammatory cytokines IL-1 alpha, IL-1 beta, IL-6, and TNF-alpha impart neuroprotection to an excitotoxin through distinct pathways J Immunol 1999 163 7 3963 3968 10490998

[B11] Diem R Hobom M Grotsch P Kramer B Bahr M Interleukin-1 beta protects neurons via the interleukin-1 (IL-1) receptor-mediated Akt pathway and by IL-1 receptor-independent decrease of transmembrane currents in vivo Mol Cell Neurosci 2003 22 4 487 500 10.1016/S1044-7431(02)00042-8 12727445

[B12] Temporin K Tanaka H Kuroda Y Okada K Yachi K Moritomo H Murase T Yoshikawa H Interleukin-1 beta promotes sensory nerve regeneration after sciatic nerve injury Neuroscience letters 2008 440 2 130 133 10.1016/j.neulet.2008.05.081 18556121

[B13] Temporin K Tanaka H Kuroda Y Okada K Yachi K Moritomo H Murase T Yoshikawa H IL-1beta promotes neurite outgrowth by deactivating RhoA via p38 MAPK pathway Biochemical and biophysical research communications 2008 365 2 375 380 10.1016/j.bbrc.2007.10.198 17996195

[B14] Edoff K Jerregard H Effects of IL-1beta, IL-6 or LIF on rat sensory neurons co-cultured with fibroblast-like cells Journal of neuroscience research 2002 67 2 255 263 10.1002/jnr.10092 11782969

[B15] Horie H Sakai I Akahori Y Kadoya T IL-1 beta enhances neurite regeneration from transected-nerve terminals of adult rat DRG Neuroreport 1997 8 8 1955 1959 10.1097/00001756-199705260-00032 9223084

[B16] Tong L Balazs R Soiampornkul R Thangnipon W Cotman CW Interleukin-1 beta impairs brain derived neurotrophic factor-induced signal transduction Neurobiology of aging 2008 29 9 1380 1393 10.1016/j.neurobiolaging.2007.02.027 17467122 PMC4052889

[B17] Soiampornkul R Tong L Thangnipon W Balazs R Cotman CW Interleukin-1beta interferes with signal transduction induced by neurotrophin-3 in cortical neurons Brain research 2008 1188 189 197 18036576 10.1016/j.brainres.2007.10.051 PMC2409119

[B18] Hendrix S Peters EM Neuronal plasticity and neuroregeneration in the skin -- the role of inflammation Journal of neuroimmunology 2007 184 1-2 113 126 10.1016/j.jneuroim.2006.11.020 17222461

[B19] Prang P Del Turco D Kapfhammer JP Regeneration of entorhinal fibers in mouse slice cultures is age dependent and can be stimulated by NT-4, GDNF, and modulators of G-proteins and protein kinase C Exp Neurol 2001 169 1 135 147 10.1006/exnr.2001.7648 11312566

[B20] Huang EJ Reichardt LF Neurotrophins: roles in neuronal development and function Annu Rev Neurosci 2001 24 677 736 10.1146/annurev.neuro.24.1.677 11520916 PMC2758233

[B21] Shornick LP De Togni P Mariathasan S Goellner J Strauss-Schoenberger J Karr RW Ferguson TA Chaplin DD Mice deficient in IL-1beta manifest impaired contact hypersensitivity to trinitrochlorobenzone The Journal of experimental medicine 1996 183 4 1427 1436 10.1084/jem.183.4.1427 8666901 PMC2192516

[B22] Hechler D Boato F Nitsch R Hendrix S Differential regulation of axon outgrowth and reinnervation by neurotrophin-3 and neurotrophin-4 in the hippocampal formation Exp Brain Res 2010 205 2 215 221 10.1007/s00221-010-2355-7 20640412

[B23] Holtje M Djalali S Hofmann F Munster-Wandowski A Hendrix S Boato F Dreger SC Grosse G Henneberger C Grantyn R A 29-amino acid fragment of Clostridium botulinum C3 protein enhances neuronal outgrowth, connectivity, and reinnervation FASEB J 2009 23 4 1115 1126 10.1096/fj.08-116855 19047066

[B24] Schmitt KR Boato F Diestel A Hechler D Kruglov A Berger F Hendrix S Hypothermia-induced neurite outgrowth is mediated by tumor necrosis factor-alpha Brain Pathol 2010 20 4 771 779 20070303 10.1111/j.1750-3639.2009.00358.x PMC8094716

[B25] Wolf SA Fisher J Bechmann I Steiner B Kwidzinski E Nitsch R Neuroprotection by T-cells depends on their subtype and activation state Journal of neuroimmunology 2002 133 1-2 72 80 10.1016/S0165-5728(02)00367-3 12446010

[B26] Steup A Lohrum M Hamscho N Savaskan NE Ninnemann O Nitsch R Fujisawa H Puschel AW Skutella T Sema3C and netrin-1 differentially affect axon growth in the hippocampal formation Molecular and cellular neurosciences 2000 15 2 141 155 10.1006/mcne.1999.0818 10673323

[B27] Schmitt KR Kern C Lange PE Berger F Abdul-Khaliq H Hendrix S S100B modulates IL-6 release and cytotoxicity from hypothermic brain cells and inhibits hypothermia-induced axonal outgrowth Neurosci Res 2007 59 1 68 73 10.1016/j.neures.2007.05.011 17604861

[B28] Golz G Uhlmann L Ludecke D Markgraf N Nitsch R Hendrix S The cytokine/neurotrophin axis in peripheral axon outgrowth Eur J Neurosci 2006 24 10 2721 2730 10.1111/j.1460-9568.2006.05155.x 17156198

[B29] Sairanen TR Lindsberg PJ Brenner M Siren AL Global forebrain ischemia results in differential cellular expression of interleukin-1beta (IL-1beta) and its receptor at mRNA and protein level J Cereb Blood Flow Metab 1997 17 10 1107 1120 9346436 10.1097/00004647-199710000-00013

[B30] Giulian D Baker TJ Shih LC Lachman LB Interleukin 1 of the central nervous system is produced by ameboid microglia J Exp Med 1986 164 2 594 604 10.1084/jem.164.2.594 3487617 PMC2188228

[B31] Shaftel SS Griffin WS O'Banion MK The role of interleukin-1 in neuroinflammation and Alzheimer disease: an evolving perspective J Neuroinflammation 2008 5 7 10.1186/1742-2094-5-7 18302763 PMC2335091

[B32] Licastro F Pedrini S Caputo L Annoni G Davis LJ Ferri C Casadei V Grimaldi LM Increased plasma levels of interleukin-1, interleukin-6 and alpha-1-antichymotrypsin in patients with Alzheimer's disease: peripheral inflammation or signals from the brain? Journal of neuroimmunology 2000 103 1 97 102 10.1016/S0165-5728(99)00226-X 10674995

[B33] Di Bona D Plaia A Vasto S Cavallone L Lescai F Franceschi C Licastro F Colonna-Romano G Lio D Candore G Association between the interleukin-1beta polymorphisms and Alzheimer's disease: a systematic review and meta-analysis Brain Res Rev 2008 59 1 155 163 10.1016/j.brainresrev.2008.07.003 18675847

[B34] Licastro F Pedrini S Ferri C Casadei V Govoni M Pession A Sciacca FL Veglia F Annoni G Bonafe M Gene polymorphism affecting alpha1-antichymotrypsin and interleukin-1 plasma levels increases Alzheimer's disease risk Ann Neurol 2000 48 3 388 391 10.1002/1531-8249(200009)48:3<388::AID-ANA16>3.0.CO;2-G 10976648

[B35] Forlenza OV Diniz BS Talib LL Mendonca VA Ojopi EB Gattaz WF Teixeira AL Increased serum IL-1beta level in Alzheimer's disease and mild cognitive impairment Dement Geriatr Cogn Disord 2009 28 6 507 512 10.1159/000255051 19996595

[B36] Smorodchenko A Wuerfel J Pohl EE Vogt J Tysiak E Glumm R Hendrix S Nitsch R Zipp F Infante-Duarte C CNS-irrelevant T-cells enter the brain, cause blood-brain barrier disruption but no glial pathology Eur J Neurosci 2007 26 6 1387 1398 10.1111/j.1460-9568.2007.05792.x 17880383

[B37] Fawcett J Molecular control of brain plasticity and repair Prog Brain Res 2009 175 501 509 19660677 10.1016/S0079-6123(09)17534-9

[B38] Fitch MT Silver J CNS injury, glial scars, and inflammation: Inhibitory extracellular matrices and regeneration failure Exp Neurol 2008 209 2 294 301 10.1016/j.expneurol.2007.05.014 17617407 PMC2268907

[B39] Schwartz M Kipnis J Model of acute injury to study neuroprotection Methods Mol Biol 2007 399 41 53 10.1007/978-1-59745-504-6_4 18309924

[B40] Gibson RM Rothwell NJ Le Feuvre RA CNS injury: the role of the cytokine IL-1 Vet J 2004 168 3 230 237 10.1016/j.tvjl.2003.10.016 15501140

[B41] Lentz SI Knudson CM Korsmeyer SJ Snider WD Neurotrophins support the development of diverse sensory axon morphologies J Neurosci 1999 19 3 1038 1048 9920667 10.1523/JNEUROSCI.19-03-01038.1999 PMC6782147

[B42] Patel TD Jackman A Rice FL Kucera J Snider WD Development of sensory neurons in the absence of NGF/TrkA signaling in vivo Neuron 2000 25 2 345 357 10.1016/S0896-6273(00)80899-5 10719890

[B43] Patel TD Kramer I Kucera J Niederkofler V Jessell TM Arber S Snider WD Peripheral NT3 signaling is required for ETS protein expression and central patterning of proprioceptive sensory afferents Neuron 2003 38 3 403 416 10.1016/S0896-6273(03)00261-7 12741988

[B44] Goldberg JL Espinosa JS Xu Y Davidson N Kovacs GT Barres BA Retinal ganglion cells do not extend axons by default: promotion by neurotrophic signaling and electrical activity Neuron 2002 33 5 689 702 10.1016/S0896-6273(02)00602-5 11879647

[B45] Fryer RH Kaplan DR Kromer LF Truncated trkB receptors on nonneuronal cells inhibit BDNF-induced neurite outgrowth in vitro Exp Neurol 1997 148 2 616 627 10.1006/exnr.1997.6699 9417837

[B46] Bosco A Linden R BDNF and NT-4 differentially modulate neurite outgrowth in developing retinal ganglion cells J Neurosci Res 1999 57 6 759 769 10.1002/(SICI)1097-4547(19990915)57:6<759::AID-JNR1>3.0.CO;2-Y 10467247

[B47] Lykissas MG Batistatou AK Charalabopoulos KA Beris AE The role of neurotrophins in axonal growth, guidance, and regeneration Curr Neurovasc Res 2007 4 2 143 151 10.2174/156720207780637216 17504212

[B48] Heimrich B Frotscher M Slice cultures as a model to study entorhinal-hippocampal interaction Hippocampus 1993 3 Spec No 11 17 8287089

[B49] Noraberg J Poulsen FR Blaabjerg M Kristensen BW Bonde C Montero M Meyer M Gramsbergen JB Zimmer J Organotypic hippocampal slice cultures for studies of brain damage, neuroprotection and neurorepair Curr Drug Targets CNS Neurol Disord 2005 4 4 435 452 10.2174/1568007054546108 16101559

[B50] Hechler D Nitsch R Hendrix S Green-fluorescent-protein-expressing mice as models for the study of axonal growth and regeneration in vitro Brain Res Rev 2006 52 1 160 169 10.1016/j.brainresrev.2006.01.005 16497382

[B51] Heppner FL Skutella T Hailer NP Haas D Nitsch R Activated microglial cells migrate towards sites of excitotoxic neuronal injury inside organotypic hippocampal slice cultures Eur J Neurosci 1998 10 10 3284 3290 10.1046/j.1460-9568.1998.00379.x 9786222

[B52] Eyupoglu IY Savaskan NE Brauer AU Nitsch R Heimrich B Identification of neuronal cell death in a model of degeneration in the hippocampus Brain Res Brain Res Protoc 2003 11 1 1 8 10.1016/S1385-299X(02)00186-1 12697257

[B53] Lee SC Dickson DW Brosnan CF Interleukin-1, nitric oxide and reactive astrocytes Brain Behav Immun 1995 9 4 345 354 10.1006/brbi.1995.1032 8903851

[B54] Feder LS Laskin DL Regulation of hepatic endothelial cell and macrophage proliferation and nitric oxide production by GM-CSF, M-CSF, and IL-1 beta following acute endotoxemia J Leukoc Biol 1994 55 4 507 513 8145021

[B55] Giulian D Lachman LB Interleukin-1 stimulation of astroglial proliferation after brain injury Science 1985 228 4698 497 499 10.1126/science.3872478 3872478

[B56] Giulian D Woodward J Young DG Krebs JF Lachman LB Interleukin-1 injected into mammalian brain stimulates astrogliosis and neovascularization J Neurosci 1988 8 7 2485 2490 2470873 10.1523/JNEUROSCI.08-07-02485.1988 PMC6569517

[B57] Giulian D Young DG Woodward J Brown DC Lachman LB Interleukin-1 is an astroglial growth factor in the developing brain J Neurosci 1988 8 2 709 714 3257519 10.1523/JNEUROSCI.08-02-00709.1988 PMC6569312

[B58] Marschinke F Stromberg I Dual effects of TNFalpha on nerve fiber formation from ventral mesencephalic organotypic tissue cultures Brain Res 2008 1215 30 39 18482714 10.1016/j.brainres.2008.03.070 PMC2586674

[B59] Hakkoum D Stoppini L Muller D Interleukin-6 promotes sprouting and functional recovery in lesioned organotypic hippocampal slice cultures J Neurochem 2007 100 3 747 757 10.1111/j.1471-4159.2006.04257.x 17144903

[B60] Lindholm D Heumann R Meyer M Thoenen H Interleukin-1 regulates synthesis of nerve growth factor in non-neuronal cells of rat sciatic nerve Nature 1987 330 6149 658 659 10.1038/330658a0 3317065

[B61] Friedman WJ Larkfors L Ayer-LeLievre C Ebendal T Olson L Persson H Regulation of beta-nerve growth factor expression by inflammatory mediators in hippocampal cultures J Neurosci Res 1990 27 3 374 382 10.1002/jnr.490270316 2129046

[B62] Gadient RA Cron KC Otten U Interleukin-1 beta and tumor necrosis factor-alpha synergistically stimulate nerve growth factor (NGF) release from cultured rat astrocytes Neurosci Lett 1990 117 3 335 340 10.1016/0304-3940(90)90687-5 2094822

[B63] Ho A Blum M Regulation of astroglial-derived dopaminergic neurotrophic factors by interleukin-1 beta in the striatum of young and middle-aged mice Exp Neurol 1997 148 1 348 359 10.1006/exnr.1997.6659 9398477

[B64] Albrecht PJ Dahl JP Stoltzfus OK Levenson R Levison SW Ciliary neurotrophic factor activates spinal cord astrocytes, stimulating their production and release of fibroblast growth factor-2, to increase motor neuron survival Exp Neurol 2002 173 1 46 62 10.1006/exnr.2001.7834 11771938

[B65] Mrak RE Griffin WS Interleukin-1, neuroinflammation, and Alzheimer's disease Neurobiology of aging 2001 22 6 903 908 10.1016/S0197-4580(01)00287-1 11754997

[B66] Pineau I Lacroix S Proinflammatory cytokine synthesis in the injured mouse spinal cord: multiphasic expression pattern and identification of the cell types involved J Comp Neurol 2007 500 2 267 285 10.1002/cne.21149 17111361

[B67] Lu K Cho CL Liang CL Chen SD Liliang PC Wang SY Chen HJ Inhibition of the MEK/ERK pathway reduces microglial activation and interleukin-1-beta expression in spinal cord ischemia/reperfusion injury in rats J Thorac Cardiovasc Surg 2007 133 4 934 941 10.1016/j.jtcvs.2006.11.038 17382630

[B68] Akuzawa S Kazui T Shi E Yamashita K Bashar AH Terada H Interleukin-1 receptor antagonist attenuates the severity of spinal cord ischemic injury in rabbits J Vasc Surg 2008 48 3 694 700 10.1016/j.jvs.2008.04.011 18572364

[B69] Guo JS Zeng YS Li HB Huang WL Liu RY Li XB Ding Y Wu LZ Cai DZ Cotransplant of neural stem cells and NT-3 gene modified Schwann cells promote the recovery of transected spinal cord injury Spinal Cord 2007 45 1 15 24 10.1038/sj.sc.3101943 16773039

[B70] Johnson PJ Parker SR Sakiyama-Elbert SE Controlled release of neurotrophin-3 from fibrin-based tissue engineering scaffolds enhances neural fiber sprouting following subacute spinal cord injury Biotechnol Bioeng 2009 104 6 1207 1214 10.1002/bit.22476 19603426 PMC2780336

[B71] Shumsky JS Tobias CA Tumolo M Long WD Giszter SF Murray M Delayed transplantation of fibroblasts genetically modified to secrete BDNF and NT-3 into a spinal cord injury site is associated with limited recovery of function Exp Neurol 2003 184 1 114 130 10.1016/S0014-4886(03)00398-4 14637085

[B72] Funakoshi H Frisen J Barbany G Timmusk T Zachrisson O Verge VM Persson H Differential expression of mRNAs for neurotrophins and their receptors after axotomy of the sciatic nerve J Cell Biol 1993 123 2 455 465 10.1083/jcb.123.2.455 8408225 PMC2119843

[B73] Lei L Parada LF Transcriptional regulation of Trk family neurotrophin receptors Cell Mol Life Sci 2007 64 5 522 532 10.1007/s00018-006-6328-8 17192812 PMC11138452

[B74] Schmitt KR Kern C Berger F Ullrich O Hendrix S Abdul-Khaliq H Methylprednisolone attenuates hypothermia- and rewarming-induced cytotoxicity and IL-6 release in isolated primary astrocytes, neurons and BV-2 microglia cells Neurosci Lett 2006 404 3 309 314 10.1016/j.neulet.2006.05.064 16860472

